# Effect of Plasma-Activated Solution Treatment on Cell Biology of *Staphylococcus aureus* and Quality of Fresh Lettuces

**DOI:** 10.3390/foods10122976

**Published:** 2021-12-03

**Authors:** Jianying Zhao, Jing Qian, Hong Zhuang, Ji Luo, Mingming Huang, Wenjing Yan, Jianhao Zhang

**Affiliations:** 1 National Center of Meat Quality and Safety Control, Collaborative Innovation Center of Meat Production and Processing, Quality and Safety Control, College of Food Science and Technology, Nanjing Agricultural University, Nanjing 210095, China; 2015208026@njau.edu.cn (J.Z.); 2019208002@njau.edu.cn (J.Q.); ywj1103@njau.edu.cn (W.Y.); 2 Quality and Safety Assessment Research Unit, U.S. National Poultry Research Center, USDA-ARS, 950 College Station Road, Athens, GA 30605, USA; hong.zhuang@ars.usda.gov; 3 College of Life Science, Anhui Normal University, Wuhu 241000, China; luoji2019336@ahnu.edu.cn; 4 College of Food Science and Engineering, Shandong Agricultural University, Tai’an 271018, China; huangmm2019@sdau.edu.cn

**Keywords:** reactive oxygen species, peptidoglycan, cell membrane, DNA, lettuces

## Abstract

This study aimed to investigate effects of plasma-activated solution (PAS) on the cell biology of *Staphylococcus aureus* and qualities of fresh lettuce leaves. PAS was prepared by dielectric barrier discharge plasma and incubated with *S. aureus* for 10–30 min or with lettuces for 10 min. Effects on cell biology were evaluated with microscopic images, cell integrity, and chemical modification of cellular components. Effects on lettuce quality were estimated with the viable microbial counts, color, contents of vitamin C and chlorophyll, and surface integrity. PAS reduced *S. aureus* population by 4.95-log and resulted in increased cell membrane leakage. It also resulted in increased contents of reactive oxygen species in cells, C=O bonds in peptidoglycan, and 8-hydroxydeoxyguanosine content in cellular DNA, and reduced ratios of unsaturated/saturated fatty acids in the cell membrane. PAS treatment reduced bacterial load on fresh lettuce and had no negative effects on the quality. Data suggest that PAS can be used for the disinfection of ready-to-eat fresh vegetables.

## 1. Introduction

Cold plasma sterilization technology has attracted intensive attention in recent years. Plasma is the fourth state of matter. In plasma, there are a variety of reactive species such as ions, electrons, free radicals, and neutral particles [1]. When a liquid is exposed to plasma, the reactive species generated in the gas phase are transferred to the liquid phase, including a series of reactive oxygen and nitrogen species [2]. The liquid treated with plasma is called as ‘plasma-activated solution’ (PAS), which has the following characteristics: low pH, high oxidation-reduction potential, rich in reactive oxygen and nitrogen species (H_2_O_2_, O_3_, NO_x_) [3,4], and appropriate for non-thermal treatments of food.

Reactive oxygen derivatives generated in plasma-activated solutions have been proved to possess significant antimicrobial activity against *Staphylococcus aureus* (*S. aureus*) and *Escherichia coli* (*E. coli*), the two most common foodborne pathogens [5,6]. Compared with *E. coli*, *S. aureus* is a Gram-positive (G^+^) bacterium and is less sensitive to the reactive oxygen derivatives due to its specific cell wall. The cell wall of *S. aureus* is composed of a peptidoglycan (PG) layer with a thickness of 20–80 nm [7,8]. Previously published studies on PAS mainly focused on its physicochemical properties and antibacterial activity [9,10]. However, the specific effects of PAS on the cell biology of *S. aureus* remain poorly understood.

The consumption of ready-to-eat vegetables with minimal processing such as lettuces has significantly increased due to the abundant amount of vitamins and minerals. However, contaminated minimally processed vegetables are responsible for many outbreaks of food poisoning and food-related infections [11]. The development of effective decontamination methods to reduce food-borne diseases are essential and urgent. Chemical sanitizers, such as ozone and chlorine, are widely used for decontamination in the fresh produce industry, but possible by-products produced by these two chemical sanitizers raise safety and environmental concerns [12]. Therefore, there is strong interest in developing a novel sanitizer to reduce microbial contamination and maintain the quality and the shelf life of minimally processed vegetables.

Thus, the objective of the present study was to investigate effects of PAS on cell biology, including cell wall, cell membrane, and DNA of treated G^+^ bacteria using *S. aureus* as a model. Specific investigations of the oxidative damages included: (1) the erosion of peptidoglycan layer of *S. aureus* cell wall; (2) lipid peroxidation in cell membranes; (3) the oxidative damage of intracellular DNA. In addition, effects of PAS as a non-thermal sanitizer on fresh lettuces were also evaluated to demonstrate its effectiveness in microbial inactivation and quality retention of fresh vegetables in practice. Results from this study could help further understand the mechanism of PAS inactivation of G^+^ bacteria and provide useful information about the application of PAS for the non-thermal treatment of fresh produce.

## 2. Materials and Methods

### 2.1. Preparation of Plasma-Activated Solution (PAS) and Sample Treatments

**Figure 1:**
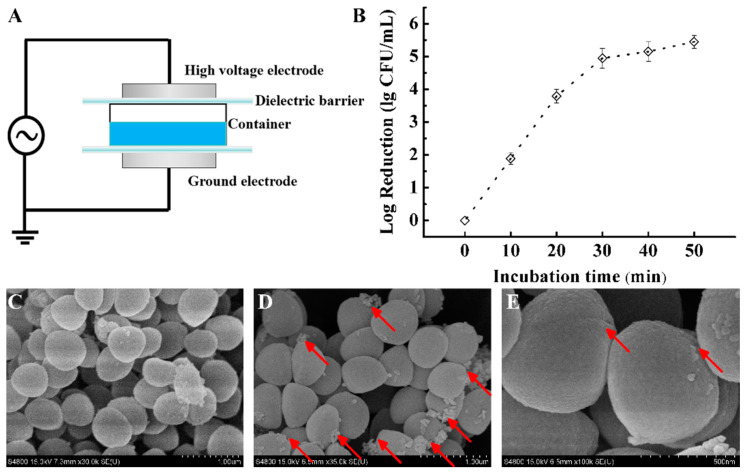
(**A**) Schematic diagram of a dielectric barrier discharge plasma system used to prepare plasma-activated solution. (**B**) Effect of incubation time on inactivation of *S. aureus* by plasma-activated solution. (**C**) SEM images of S. aureus at time 0. (**D**,**E**) SEM images of *S. aureus* treated with plasma-activated solution for 30 min.

Cold plasma treatment was carried out using the dielectric barrier discharge (DBD) device (Phenix Technologies Inc., Accident, MD, USA) as exhibited in Figure 1A. For PAS preparation, 100 mL of solution was transferred into a polypropylene container and the container was sealed with the polyethylene film. Samples were placed between two circular aluminum electrodes (diameter was 15 cm, the distance between the two electrodes was 40 mm). There was a dielectric barrier layer between the electrode and the sample. Power supply parameters for treatments were: 60 kV voltage and 50 Hz frequency. Air was used as the working gas.

To improve the antibacterial efficiency of PAS, 800 µL hydrogen peroxide (H_2_O_2_, the concentration was 3%) was added to 100-mL deionized water, which was determined according to our preliminary experiments (the solution without plasma treatment had no obvious effect on bacterial cells). The solution was treated by DBD plasma for 4 min and stored at room temperature for 6 h before the effects were evaluated (the pH value of PAS was around 2.28). For the effect on the cell biology of *S. aureus*, PAS was mixed with bacterial suspension in a 10:1 ratio (*v*/*v*) and incubated at room temperature for 0 (control), 10, 20, or 30 min before bacteria were sampled for different analyses. For lettuce experiments, fresh lettuces were soaked in PAS for 0 (control) or 10 min before quality assessments were conducted.

### 2.2. Antibacterial Action of PAS

#### 2.2.1. Bacterial Cell Enumeration

*Staphylococcus aureus* (*S. aureus*, ATCC6538) was chosen as a model bacterium to assess the antibacterial action of PAS. *S. aureus* was cultured at 37 °C for 18 h in the nutrient broth to achieve log-phase. The cultured bacteria were washed using saline (0.85%, *w*/*v*) three times by centrifugation and diluted to 10^6^ CFU/mL. Then, the bacteria suspension was incubated with PAS for different times (0, 10, 20, or 30 min). After incubation, 0.5 mL of the mixed suspension was taken, diluted serially with saline solution, and 100 µL-dilution was plated on mannitol high salt agar. The bacterial colonies formed on the agar plate were counted after incubation at 37 °C for 24 h.

#### 2.2.2. Cell Morphology Image

Changes in cell morphology of treated *S. aureus* were observed using scanning electron microscopy (SEM). Samples after treatments were washed with 0.01 M of phosphate-buffered saline (PBS, pH 7.2) five times and fixed with 2.5% glutaraldehyde at 4 °C overnight. After the fixation, samples were washed with PBS and deionized water three times each and then dehydrated in graded ethanol concentrations of 30, 50, 70, 85, 95, and 100%. The samples were freeze-dried and assessed visually using the S4800 SEM (Hitachi Co. Ltd., Tokyo, Japan) [13].

#### 2.2.3. Cell Histology Image

Transmission electron microscopy (TEM) was used to examine the effect of PAS treatment on the cell histology of *S. aureus*. Samples, after being washed as described in Section 2.2.2, were fixed in 2.5% glutaraldehyde for at least 2 h, washed, and further prepared as described by Li et al. [14]. Bacterial cells were observed using a JEM-2100 TEM (JEOL Ltd., Tokyo, Japan).

#### 2.2.4. Cell Membrane Integrity

The membrane integrity of PAS-treated *S. aureus* cells was indicated with the measurements of the release of intracellular components, DNA, proteins, and K^+^. The release of DNA and protein was indicated by changes in the absorbance at 260 and 280 nm, respectively, using an ultraviolet absorption spectrometer (UV-2600/2700, Shimadzu, Japan). Untreated and PAS-treated bacterial suspensions were centrifuged at 10,000× *g* for 10 min, and the supernatants were collected for analysis. The release of DNA was calculated using the formula Leakage Ratio = A260 (PAS-treated)/A260 (untreated), and the release of protein using Leakage Ratio = A280 (PAS-treated)/A280 (untreated) [14,15,16]. The release of K^+^ was detected according to the instruction of the K^+^ detection kit (Wuhan Elarite Biological Technology Co., Ltd., Wuhan, China). Results expressed as: Leakage Ratio = K^+^ concentration (PAS-treated)/K^+^ concentration (untreated).

### 2.3. Oxidative Damages to S. aureus Cells

#### 2.3.1. ROS in *S. aureus* Cells

To detect the intracellular ROS level, 2′,7′-dichlorodihydrofluorescein diacetate (H_2_DCFDA, Aladdin, Shanghai, China) was used as a fluorescent probe. First, 100 μL of washed bacterial suspension (see Section 2.2.2) was mixed with 100 μL of 40-μM H_2_DCFDA and incubated at 37 °C for 30 min in the dark. Then, the solution was washed again and resuspended in PBS at 37 °C for 15 min. The fluorescent analysis was performed by a microplate reader (SpectraMax M2e, Molecular Devices, Sunnyvale, CA, USA) at 495 nm (excitation) and 525 nm (emission) [17].

#### 2.3.2. Peptidoglycan in Cell Wall

Changes in different atomic contents in peptidoglycan (PA) in bacterial cell walls after PAS treatment were investigated by X-ray photoelectron spectroscopy (XPS, Thermo Scientific). Bacteria suspension after treatment was washed using saline (0.85 %, *w*/*v*) three times. A non-monochromatized Al Kα X-ray source was used at 15 kV and 20 mA, and all spectra were achieved at a take-off angle of 15° relative to the sample [18].

#### 2.3.3. Fatty Acids in Cell Membrane

Bacterial suspensions treated with/without PAS were centrifuged to collect bacteria pellets. After the pellets were washed three times with PBS (0.01 M, pH 7.2), 40 mg of them were saponified and methylated and the fatty acid methyl esters were extracted according to the method reported by Wang, et al. [19]. One mL of the organic phase was transferred to a vial for subsequent Gas chromatographic (GC) analysis.

GC analysis of fatty acids was carried out with 37 kinds of fatty acid methyl esters as external standards. The GC spectrometer (2010 Plus, Shimadzu Co., Tokyo, Japan) was equipped with a capillary column (SPTM-2560, 100 m × 0.25 mm × 0.2 μm, Shimadzu Co., Tokyo, Japan). Helium was used as a carrier gas with a flow rate of 9.6 mL/min and in a split mode of 10:1 [17]. The initial pressure of the injector was 527 kPa, and the temperature of both the injector and flame ion detector (FID) was 260 °C. The temperature program was started at 100 °C, followed by an increase of 4 °C/min to 260 °C, and held for 30 min. The results were expressed as a ratio of total unsaturated fatty acids (area)/total saturated fatty acids (area).

#### 2.3.4. DNA

A by-product of DNA damage in prokaryotic cells, 8-hydroxydeoxyguanosine (8-OHdG), is generated by the oxidative modification of guanine residues [20]. Thus, 8-OHdG is commonly used as an indicator for oxidative damages in prokaryotic DNA. After PAS treatment, bacterial suspensions were centrifuged and suspended in PBS. DNA was extracted by the DNA Extraction Kit (Qiagen, Duesseldorf, Germany) according to the manufacturer’s instruction. OxiSelect™ Oxidative DNA Damage ELISA Kit (Cell Biolabs, USSA) was applied to determine the changes in the concentration of 8-OHdG. For the electrophoresis analysis, DNA samples were loaded on a 1% agarose gel and strained by GoldView [21].

### 2.4. Qualities of Fresh Lettuces

Fresh lettuces were purchased from the local supermarket in Nanjing, China. Samples without mechanical damage were immersed in PAS for 10 min, and changes in the quality of lettuce before and after plasma-activated solution treatment were investigated. The lettuce treated with deionized water instead was used as the control. A colorimeter (Color difference meter, CR-400, Konica-Minolta, Tokyo, Japan) was used to evaluate changes in the color of lettuce leaves after treatments. The total viable bacteria count, the content of vitamin C, and chlorophyll were determined according to the methods reported in our previously published study [22]. For SEM images of lettuce leaves, samples after treatment were cut into 2 mm × 2 mm and fixed in 2.5% glutaraldehyde solution. After the fixation, samples were prepared according to Section 2.2.2.

### 2.5. Statistical Analysis

Experiments were repeated three times independently with duplicate samples. Statistical analysis was performed by one-way analysis of variance (ANOVA), and the significant differences between mean values were identified using Duncan’s test in SPSS software with a significance level of *p* < 0.05.

## 3. Results and Discussion

### 3.1. Effect on S. aureus Survival

The antibacterial efficacy of PAS was investigated against *S. aureus* after various times of incubation (Figure 1B). A substantial reduction (4.95 log CFU/mL) was observed when the duration of the bacterial exposure to PAS increased from 0 to 30 min. The reduction in the bacterium exhibited a flat tendency with further increasing incubation time. These results demonstrated that the PAS was very effective in the inactivation of G^+^ pathogen *S. aureus* in liquid media. A similar relationship between treatment time and bacterial survival was also observed when *S. aureus* cells were treated directly under plasma [8]. The antimicrobial efficacy of the PAS could be attributed to reactive oxygen species (ROS) formed or dissolved in it during plasma generation. It has been proved that there are numerous ROSs in the PAS, such as long-lived species hydrogen peroxide (H_2_O_2_), nitrite (NO_2_^−^), nitrate (NO_3_^−^), and ozone (O_3_), and short-lived species hydrogen radicals (OH^•^), superoxide (O_2_^−^), peroxynitrites (ONOO^−^), and other radicals [23,24]. Han et al. [8] attributed the number of reduction in treated *S. aureus* cells to ROSs generated in the atmospheric plasma.

### 3.2. Effect on S. aureus Morphology

SEM images showed that there were apparent changes in the morphology of *S. aureus* cells after PAS treatment for 30 min (Figure 1C–E). Compared with untreated bacteria, the surface of bacteria after PAS treatment was rough and shrunken. In some cases, the hole was formed on cell surfaces. Similar results were also found when *S. aureus* cells were treated with atmospheric plasma directly [8]. Our results demonstrated that PAS treatment could also cause damages to the appearance of *S. aureus* cells.

### 3.3. Effect on Cell Histology of S. aureus

**Figure 2:**
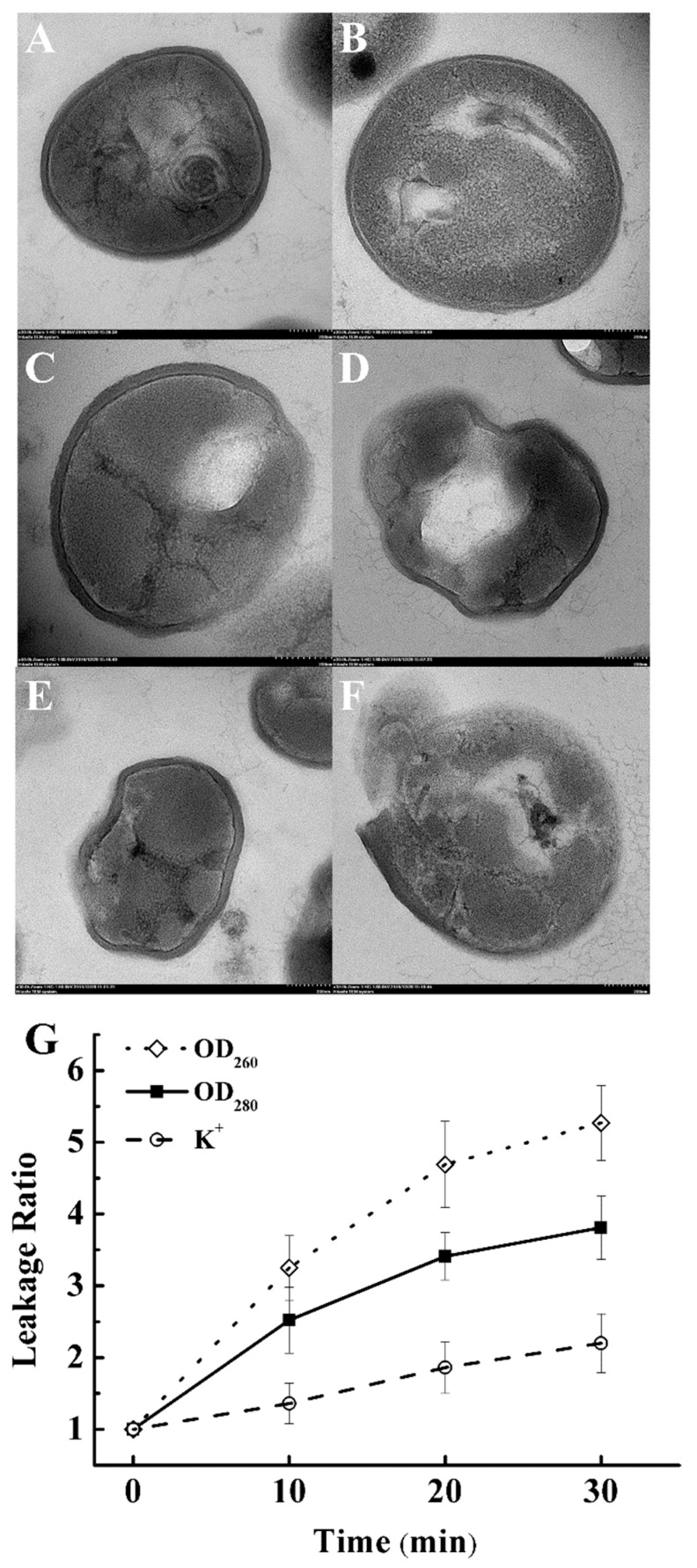
TEM images of *S. aureus* of the control (**A**,**B**) or treated with plasma-activated solution (**C**–**F**) for 30 min. (**G**) The leakage of intracellular components DNA, proteins, and K^+^, during incubation.

In addition to cell morphology, we also evaluated the effect of PAS treatment on cell histology of *S. aureus* using TEM images. A G^+^ bacterium possesses a thick cell wall, in which the peptidoglycan (PG) layer accounts for 60–95% [7]. Therefore, the effect of PAS treatment on the cell PG layer was evaluated in TEM images. TEM was applied to observe the changes in the PG layer after PAS treatment. Figure 2A,B illustrates the cell histology of the control group (without PAS treatment). The bacteria were elliptical with a clear cell outline. The cell wall was closely connected with the cell membrane and the cell cytoplasm looked like the images showing a normal G^+^ bacterium. After PAS treatments (Figure 2C–F), the outline of bacteria became irregular and some areas were blurry. There was a gap between the bacterial cell wall and membrane. The cytoplasm lost its integrity and some portions became blank. The leakage of cytoplasm and damage of the cell envelope was noticeable. The damaged PG layer and leaked cytoplasm further confirmed that the antioxidant system in *S. aureus* could not resist the excessive ROSs generated in PAS, which also induced further penetration of ROSs into the intracellular cytoplasm. These results demonstrate that PAS treatment does not only affect the cell morphology but also cell histology or cell integrity of G^+^ bacteria.

### 3.4. Effect on Cell Membrane Integrity

The effect of PAS on the cell membrane integrity of *S. aureus* was also evaluated by measuring leakage of intracellular components, DNA, proteins, and K^+^. Collected data showed that increasing incubation time resulted in increased leakage ratios of the intracellular components (Figure 2G). The leakage of DNA (280 nm) and protein (260 nm) increased significantly in the first 10 min. When the incubation time extended beyond 10 min, the leakage increased slightly. Meanwhile, the content of potassium ions in the supernatant also increased with the prolongation of PAS treatment time over 10 min. The leakage could be due to the damage caused by PAS to the transport function in the cell membrane, such as the Na^+^/K^+^—ATPase activity on the phospholipid bilayer of the cell membrane [25] or the increasing degree of cell rupture (Figure 2G). These data provided further evidence to indicate that PAS treatment could cause damage to the integrity of the bacterial membrane.

### 3.5. Effect on ROS Levels in S. aureus Cells

**Figure 3:**
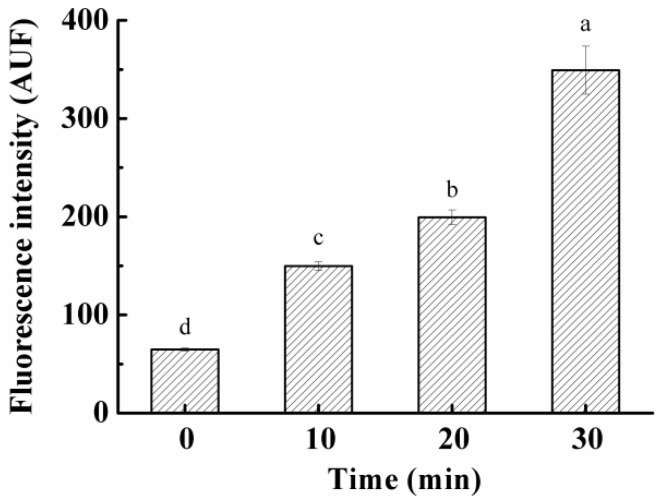
The level of intracellular ROSs of *S. aureus* after exposure to plasma-activated solution for various times. Different letters over the bars indicate a significant difference (*p* < 0.05) among incubation time.

Oxidative stress or ROS formation has been hypothesized for the inactivation of bacteria by PAS. Thus, the level of intracellular ROS was also assessed with the H_2_DCFDA method to provide evidence of ROS involvement in the present study. The fluorescence intensity increased remarkably from 65 to 350 AFU (*p* < 0.05) with increasing PAS treatment time and exhibited the incubation time-dependent behavior (Figure 3). It was similar to the behaviors in the reduction in bacterial population (Figure 1B) and the cell membrane leakage (Figure 2G) after PAS treatments. These results demonstrated that PAS treatment resulted in increased ROS levels or oxidative stress in *S. aureus* cells and indicate that PAS might inactivate G^+^ bacteria through increasing ROSs in cells or ROS-induced oxidative damages/stress.

### 3.6. Effect on Chemical Bonds of Peptidoglycan Layer

The changes in the atomic contents of PG were investigated, as indicators for oxidative damage to the cell wall of *S. aureus* by PAS treatment, using the XPS analysis. Accumulation of ROSs on the PG layer, which is the outer part of the bacteria wall, led to erosion and the formation of lesions, as shown in Table 1. The binding energy for atomic orbitals P 2p, C 1s, O 1s, and N 1s was 132.6, 399.1, 530.9, and 284 eV, respectively, in the XPS spectra. C 1s and O 1s accounted for 88.37% of the atomic percentage. During PAS treatment, the atomic percentage of orbital C 1s decreased from 65.11 to 36.91%, while the atomic percentage of orbital O 1s increased from 23.26 to 55.13%. In addition, the O/C ratio also increased from 0.36 to 1.49. C 1s is mainly related to C-H/C-C, C-O, and C=O bonds; meanwhile, O 1s is mainly related to C=O and C-OH [26,27]. Therefore, increases in the atomic O percentage could indicate that PAS treatments result in increased C=O bonds in PG. The increased O/C ratio in PG might be attributed to the ROS in PAS, especially H_2_O_2_, OH^•^, and O_3_, capable of damaging the cell wall by breaking down the bonds of PG. Mandal et al. [28] showed that ROSs could break chemical bonds in PG such as a C-O bond and cause cell wall damages, which increased the permeabilization of the cell envelope and promoted penetration of ROSs into bacteria. In our study, large changes in the ratio of O/C suggested that PAS treatments led to the substantial modification of the PG layer in the *S. aureus* cell wall. The modification may damage the integrity and functionality of the cell wall of a G^+^ bacterium and result in the inactivation of bacteria.

### 3.7. Effect on Fatty Acids in S. aureus Cell Membrane

**Figure 4:**
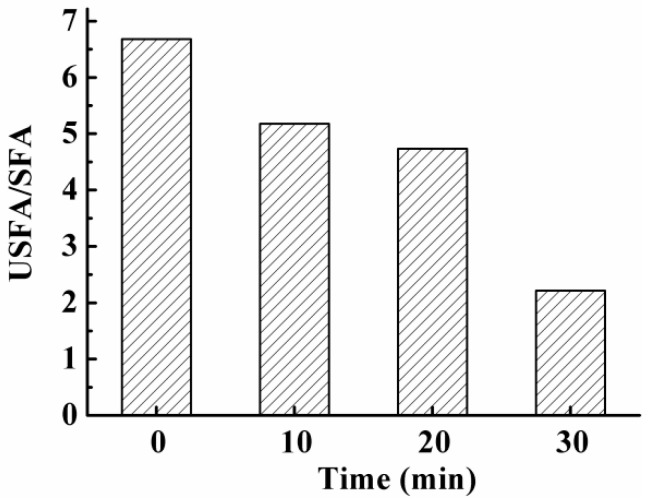
The ratio of unsaturated fatty acids (USFA) to saturated fatty acids (SFA) in the cell membrane of *S. aureus* after exposure to plasma-activated solution for various times.

Data from the leakage experiment presented above indicated that the cell membrane of *S. aureus* was either damaged or dysfunctional after PAS treatments. Lipids are the main components in a cell membrane and also the most vulnerable macromolecules to oxidation. Changes in cell membrane lipids could be a good indicator of cell membrane integrity and functionality. Therefore, in the present study, contents of unsaturated fatty acids (USFA) and saturated fatty acids (SFA) in cell lipids of *S. aureus* were determined after PAS treatment and ratios of USFA/SFA were calculated and used as indicators for the oxidative damage to the cell membrane. As displayed in Figure 4, the ratio of USFA/SFA in *S. aureus* continuously decreased from 6.7 to 2.2 with increasing incubation time, suggesting that PAS treatments resulted in the oxidation of USFAs. Compared with SFA, USFAs are much more easily attacked by ROS, such as OH^•^, and subject to lipid peroxidation [29,30]. Therefore, our data indicated that oxidative damages to the cell membrane occurred after bacteria are exposed to PAS. It is well known that the fatty acid composition in lipids directly influences the fluidity of the cell membrane [31]. An increase in the relative proportion of saturated fatty acids would lead to poor fluidity of cell membranes, which subsequently causes damage to functions of the membrane such as the maintenance of cellular morphology and permeability, message passing, and substance exchange [19].

### 3.8. Effect on DNA in Bacterial Cells

**Figure 5:**
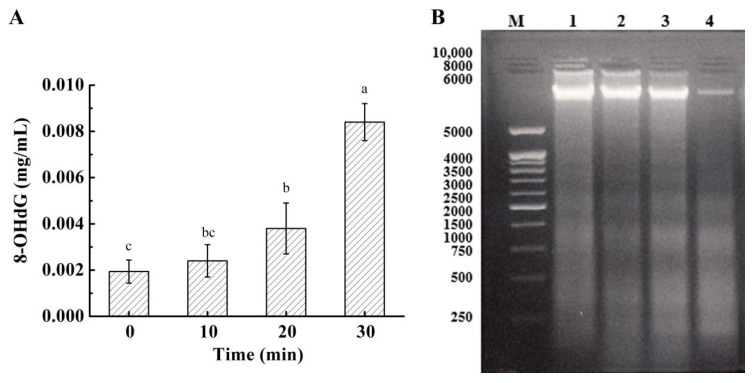
(**A**) Changes in the content of 8-OHdG in *S. aureus* after exposure to plasma-activated solution for various times. (**B**) The agarose gel of *S. aureus* DNA after plasma-activated solution treatment. M: DL 10,000 DNA Markers, 1–4: *S. aureus* DNA from samples incubated with plasma-activated solution for 30, 20, 10, and 0 min, respectively. Different letters over the bars indicate a significant difference (*p* < 0.05) among incubation time.

The oxidation of intracellular DNA is considered the most biologically significant target of oxidative attacks/damages [20]. The formation of 8-OHdG during the oxidation of deoxyguanosine (dG) has been widely used for indicating the degree of oxidatively damaged DNA [32]. Figure 5A showed the concentration of 8-OHdG measured in *S. aureus* cells after PAS treatments. It increased slowly in the first 20 min and exhibited a drastic increase after *S. aureus* cells were incubated with PAS for 30 min. Results suggested that the DNA in *S. aureus* cells is oxidized during PAS treatments and the oxidative stress/damages might have been induced by PAS in cells.

**Figure 6:**
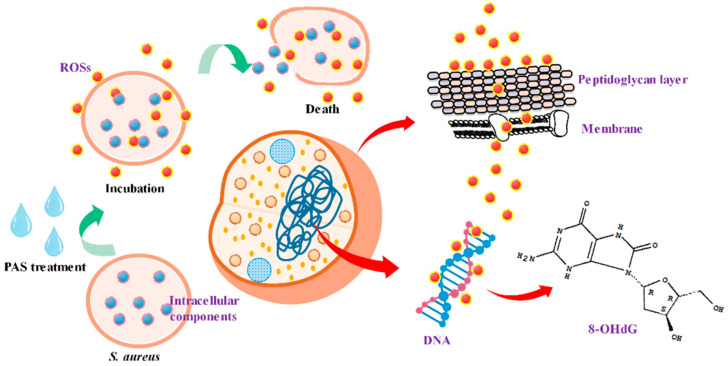
The schematic diagram for oxidative damages to *S. aureus* after plasma-activated solution treatment. *S. aureus* cell + PAS → attacked peptidoglycan layer, membrane, and DNA → cell death.

The above data were further validated with 1% agarose gel electrophoresis of DNA. The bandwidth of DNA fragments with a molecular size of near 6000 turned thicker and brighter with increasing exposure time to PAS (Figure 5B), suggesting that the DNA oxidation in *S. aureus*. It is known that reactive species involved in the oxidation of cell DNA are mainly OH^•^ (high reactivity with cellular components) [32,33], anions, and nitrite (NO_2_^•^, ONOO^−^, nitration of nucleic acids) [34]. Our data demonstrated that ROSs existed in PAS-treated G^+^ bacterial cells; the pathway is illustrated in Figure 6.

### 3.9. Effect on Quality of Fresh Lettuces

**Figure 7:**
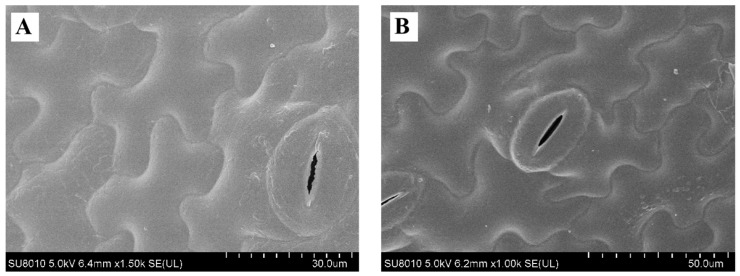
SEM images of lettuce leaves after (**A**) deionized water and (**B**) PAS treatments.

After fresh lettuce leaves were soaked in PAS for 10 min, the total viable bacterial count decreased from the initial 5.18 to 3.95 log CFU/g (Table 2), demonstrating that PAS as the sanitizer could inactivate microbiota on vegetable leaves. As observed in the SEM images (Figure 7A,B), there were no visible differences in the surface of lettuce leaves between the control and PAS treatments. The integrity of the stomata was not damaged. There were no significant differences in green color indicated with a* values, vitamin C, and chlorophyll contents between the control and PAS groups. These results suggest that PAS treatment could not only improve microbiological quality but also retain the nutrient quality with no adverse effect on the appearance of fresh vegetable leaves.

## 4. Conclusions

PAS generated by DBD plasma treatment could effectively inactivate G^+^ bacteria, damage bacterial cells, and result in the oxidation of bacterial cell walls, cell membranes, and cellular DNA. The effects of exposure time on PAS antimicrobial efficacy and damages to bacterial cell integrity were highly associated with the oxidation of different cell components during the treatment. PAS treatment did not only improve the microbiological quality of fresh leafy vegetables but also had no negative effects on their nutrient values and appearance. These results also indicated that the inactivation of G^+^ bacteria by PAS might result from the ROSs formed during plasma generation in the solution, which further oxidized components in cell walls, lipids in the cell membrane, and cellular DNA, damaged cell integrity, and led to changes in cell histology and morphology and subsequently cell death. PAS can be used as an alternative sanitizer for non-thermally disinfecting microbiota and foodborne pathogens and retaining the quality of fresh fruits and vegetables.

## Figures and Tables

**Table 1:** Changes in the atomic percentage of peptidoglycan in *S. aureus* cell wall after exposure to plasma-activated solution for various times.

Orbital	Binding Energy (eV)	Atomic Percentage (%)			
		Control	10 min	20 min	30 min
P 2p	132.6	1.64	1.79	1.87	2.27
C 1s	399.1	65.11	52.36	41.31	36.91
O 1s	530.9	23.26	36.19	48.4	55.13
N 1s	284.4	9.99	9.66	8.42	5.69
O/C	530.9/399.1	0.36	0.69	1.17	1.49

**Table 2:** Changes in the color, total viable bacteria count, vitamin C, and chlorophyll content of lettuces after plasma-activated solution treatment.

Treatment	L* (Brightness)	a* (Greenness)	b* (Yellowness)	Total Viable Bacteria Count (lg CFU/g)	Vitamin C (mg/100 g)	Chlorophyll (mg/g)
Control	58.37 ± 3.69	−11.49 ± 1.84	36.31 ± 2.97	5.18 ± 0.34	8.67 ± 0.54	1.38 ± 0.18
PAS	56.14 ± 1.28	−13.15 ± 2.37	35.47 ± 3.24	3.95 ± 0.28	8.11 ± 0.49	1.45 ± 0.25

## Data Availability

The data that support the findings of this study are available from the corresponding author upon reasonable request.
